# Oceanographic connectivity strongly restricts future range expansions of critical marine forest species

**DOI:** 10.1038/s44185-026-00123-y

**Published:** 2026-03-27

**Authors:** Jorge Assis, Eliza Fragkopoulou, Ester A. Serrão, Miguel B. Araújo

**Affiliations:** 1 Centro de Ciências do Mar do Algarve (CCMAR/CIMAR LA), Campus de Gambelas, Universidade do Algarve, Faro, Portugal; 2 Faculty of Bioscience and Aquaculture, Nord Universitet, Bodø, Norway; 3 Department of Biogeography and Global Change, National Museum of Natural Sciences, Consejo Superior de Investigaciones Científicas (CSIC), Madrid, Spain; 4 “Rui Nabeiro” Biodiversity Chair, MED – Mediterranean Institute for Agriculture, Environment and Development & CHANGE – Global Change and Sustainability Institute, University of Évora, Évora, Portugal; 5 German Centre for Integrative Biodiversity Research (iDiv), Halle-Jena-Leipzig, Leipzig, Germany

**Keywords:** Ecology, Ecology, Ocean sciences

## Abstract

Climate change is driving a global redistribution of marine biodiversity. As habitats shift, oceanographic connectivity (the transport of dispersive stages via ocean currents) becomes a critical, yet poorly understood, factor that can either facilitate or hinder species’ expansions into new areas. To quantify this influence, we developed a framework linking species distribution models with biophysical connectivity models to examine the redistribution of 467 marine forest species (seagrasses and brown macroalgae) under end-of-century climate change scenarios. Our projections show substantial habitat loss for both groups, reaching up to 50% (seagrasses) and 58% (brown macroalgae) of current habitats under higher emissions. Despite potential poleward expansions, oceanographic connectivity emerges as a major limiting factor. Accounting for average dispersal duration, range expansions are reduced by up to 38% in area (seagrasses) and 48% (macroalgae), and by up to 64% and 72% in distance, respectively. This reduction significantly increases the percentage of species facing a net loss of suitable habitat. Notably, well-defined dispersal barriers restrict expansions into highly suitable regions (e.g., the Okhotsk Sea, New Zealand, and the Arctic). Our findings underscore the need to explicitly integrate both habitat suitability and oceanographic connectivity to accurately predict marine biodiversity redistribution and inform effective conservation strategies.

## Introduction

Climate change is projected to significantly shift global marine biodiversity patterns and community structure^1–7^. While range expansions into newly suitable habitats can partially offset the negative impacts of projected range losses^1^, oceanographic connectivity may constrain the extent of future changes^4^. This connectivity is the outcome of the complex interaction between ocean current patterns and species’ dispersal potential, which can vary significantly, from a few hours to hundreds of days^5,6^. Depending on its alignment with climate shifts, oceanographic connectivity can either create barriers that isolate populations (increasing extinction risk as populations fail to track shifting conditions) or support persistence by facilitating range displacement into newly suitable habitats^4,7–9^. Despite the recognized role of ocean currents in shaping marine species’ responses to climate change, the extent to which they restrict or facilitate species redistributions remains a critical and largely unquantified gap in our understanding. This gap is especially critical for marine forest species of seagrass and brown macroalgae, critical ecosystem-restructuring species for which high-emission scenarios project drastic poleward and depth distribution shifts^10,11^ and whose dispersal relies heavily on passive transport by ocean currents^12^. Given the crucial ecological and economic services provided by these marine forests - including coastal protection, food security, and carbon sequestration^13–15^ - anticipating their potential redistribution under future climate changes has far-reaching implications for ecosystem services and conservation policy.

Current distribution models projecting future species distributions often rely on oversimplified assumptions of marine connectivity, such as unlimited dispersal to new suitable habitats, complete restriction, or distance-based criteria (see for a discussion of alternatives^16^). Overall, these assumptions fail to capture the asymmetric and highly variable nature of ocean currents, which are shaped by complex oceanographic (e.g., gyres, eddies, tidal forces, and fronts) and topographic features^17,18^. Additionally, ocean current patterns interact with species-specific dispersal potential (e.g., the duration of viable propagules) to create highly variable connectivity patterns that ultimately shape species’ ability to track shifting climates. To address these limitations, advancements in modeling now call for sophisticated biophysical models that integrate high-resolution, time-varying oceanographic data alongside relevant biological traits. By simulating the trajectory of propagules advected by ocean currents, these models offer more realistic predictions of dispersal pathways and potential settlement locations^19,20^. Importantly, in the case of marine forests, oceanographic connectivity derived from biophysical models has been shown to systematically explain patterns of population genetic differentiation^12,21,22^, providing strong empirical support for their use in capturing the influence of ocean currents on species redistributions.

In this study, we quantify the extent to which oceanographic connectivity restricts future range expansions of seagrasses and brown macroalgae across contrasting climate scenarios. We couple habitat-suitability projections from species distribution models (SDMs) with connectivity estimates from biophysical models (BMs) to calculate net distribution changes and pinpoint regions where ocean currents systematically shape barriers to dispersal. By revealing where and to what extent ocean currents limit – or facilitate – the colonization of newly suitable habitats under climate change, our approach delivers crucial insights for biodiversity assessments and management strategies that incorporate the dynamic interplay between shifting climates and complex oceanographic processes.

## Results

### Species habitat changes under future climate change

**Fig. 1:**
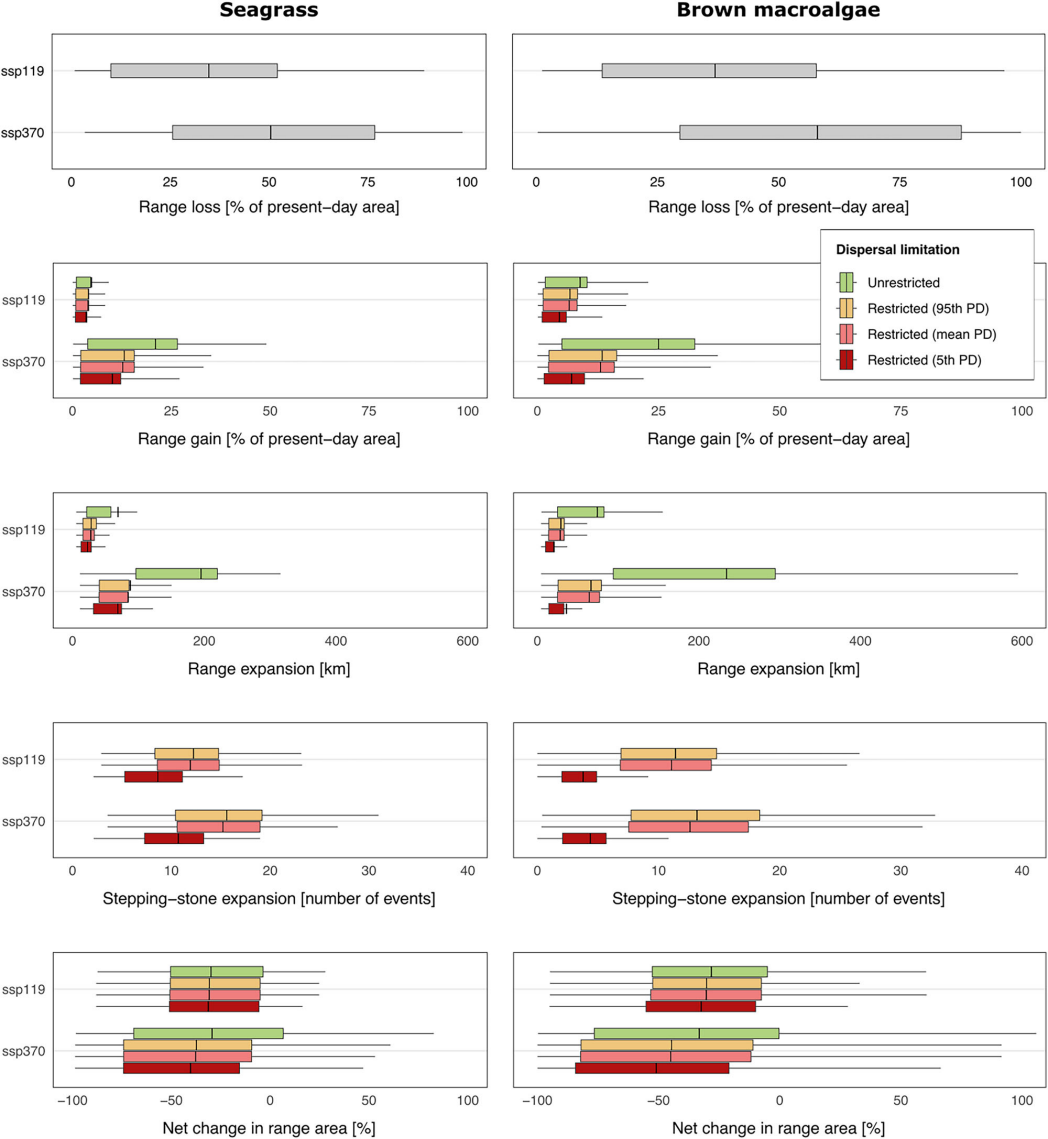
Influence of oceanographic connectivity in projected range shifts of (left column) seagrasses and (right column) brown macroalgae under contrasting Shared Socioeconomic Pathway (SSP) scenarios of climate change Unrestricted versus restricted dispersal by oceanographic connectivity to newly suitable habitats with the 5th percentile, mean, and 95th percentile of propagule duration (PD) periods of each taxonomic group. Habitat suitability loss and gain in percentage area change. Potential range expansions in distance. Number of stepping-stone connectivity events to newly suitable habitats. Net change in suitable habitats in percentage area. Solid vertical lines over boxplots depict the average of each metric. Data per species, climate scenario, and dispersal restrictions are available in Supplementary Data 2.

Higher emission scenarios intensified habitat loss, affecting brown macroalgae to a higher extent (Supplementary Data 2; LMM and Tukey tests in Supplementary Data 3). Specifically, under the lower emission scenario (SSP1-1.9), seagrasses lose 34.75% of their habitat, while brown macroalgae lose 37.01%. Under the higher emission scenario (SSP3-7.0), these losses increased to 50.39% for seagrasses and 58.22% for brown macroalgae (Fig. 1; Supplementary Data 4). Habitat gains also depended on the emission scenario, both in terms of area and expansion distance, but were independent of taxon (Supplementary Data 2–3). Under the unlimited dispersal assumption, similar area gains varied between 4.79 and 21.02%, and between 8.80 and 25.01%, for seagrass and brown algae, respectively, while expansion distances varied between 69.06 and 194.97 km, and between 74.04 and 234.22 km (SSP1-1.9 vs. SSP3-7.0; Fig. 1; Supplementary Data 4).

### Impact of oceanographic connectivity on species range shifts

Incorporating oceanographic connectivity substantially restricted dispersal potential to future suitable habitats, with no detectable differences between estimates based on the mean and 95th percentile of propagule durations (Supplementary Data 3). Thus, subsequent analysis and discussion focus solely on the results based on the 5th percentile and the mean of propagule duration. For seagrasses, incorporating oceanographic connectivity under the mean propagule-duration scenario restricted expansions by 17.78 and 39.52% in area and by 59.88 and 56.70% in distance (SSP1-1.9 vs. SSP3-7.0, respectively). Under the 5th-percentile propagule duration scenario, restrictions were stronger, with expansions reduced by 28.54 and 51.93% in area and by 66.85 and 64.77% in distance (SSP1-1.9 vs. SSP3-7.0). For brown macroalgae, connectivity under the mean propagule-duration scenario restricted expansions by 25.49 and 47.89% in area and by 59.88 and 56.70% in distance (SSP1-1.9 vs. SSP3-7.0; Fig. 1). Under the scenario of 5th percentile scenario, reductions again increased, to 48.40 and 71.75% in area and by 66.85 and 64.77% in distance (SSP1-1.9 vs. SSP3-7.0; Fig. 1).

Under restricted oceanographic connectivity, successful range expansions for seagrasses involved an average of 11.91–15.23% stepping-stone connectivity events under the mean propagule-duration scenario (SSP1-1.9 vs. SSP3-7.0), compared with the substantially lower range of 8.63–10.71 events under the 5th percentile. For brown macroalgae, the reduction was even more pronounced: expansions involved an average of 11.07–12.60 events under the mean propagule-duration scenario, but only 3.78–4.37 events under the 5th percentile (Fig. 1).

Overall, accounting for restricted dispersal under the mean propagule duration reduced the net change in suitable habitats up to 28.28% for seagrasses and 35.40% for brown macroalgae (SSP3-7.0; Fig. 1), increasing the percentage of projected species experiencing negative net changes by up to 13.16% for seagrasses and 11.99% for brown macroalgae. Under the 5th percentile, the magnitude of the impact increased further, with net changes reduced by up to 37.16% for seagrasses and 53.38% for brown macroalgae, and the percentage of projected species experiencing negative net changes increased by up to 18.42 and 21.44%, respectively.

**Fig. 2:**
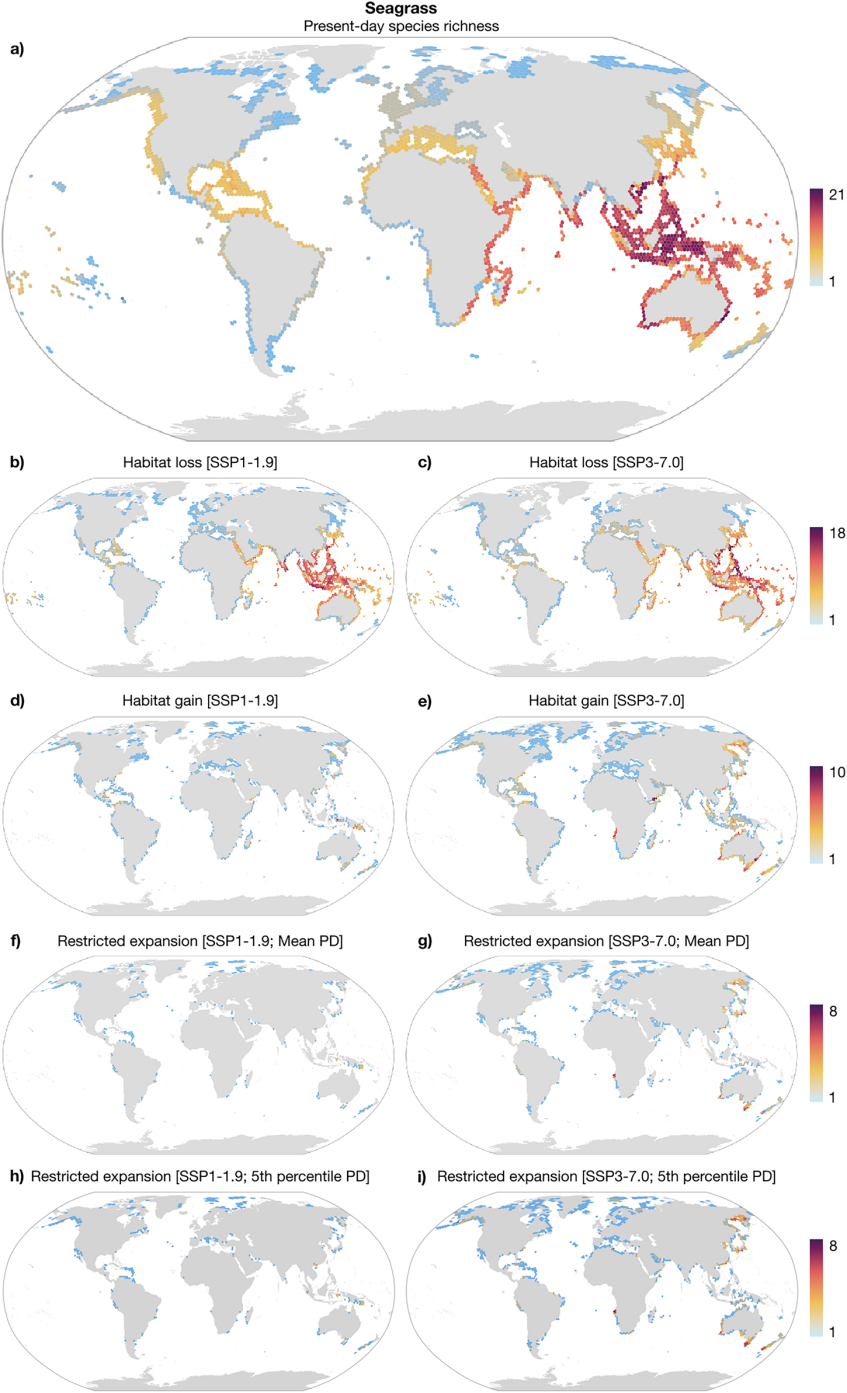
Projected range shifts of seagrasses under contrasting Shared Socioeconomic Pathway (SSP) scenarios of climate change. Present-day patterns of **a** species richness and projected habitat **b**, **c** losses and **d**, **e** gains under climate change. Regions of restricted range expansion to newly suitable habitats owing to the effect of oceanographic connectivity under the **f**, **g** mean and the **h**, **i** 5th percentile of propagule duration. Maps aggregated to a coarser resolution of 150 km for better visualization (refer to data availability statement for high-resolution maps).

**Fig. 3:**
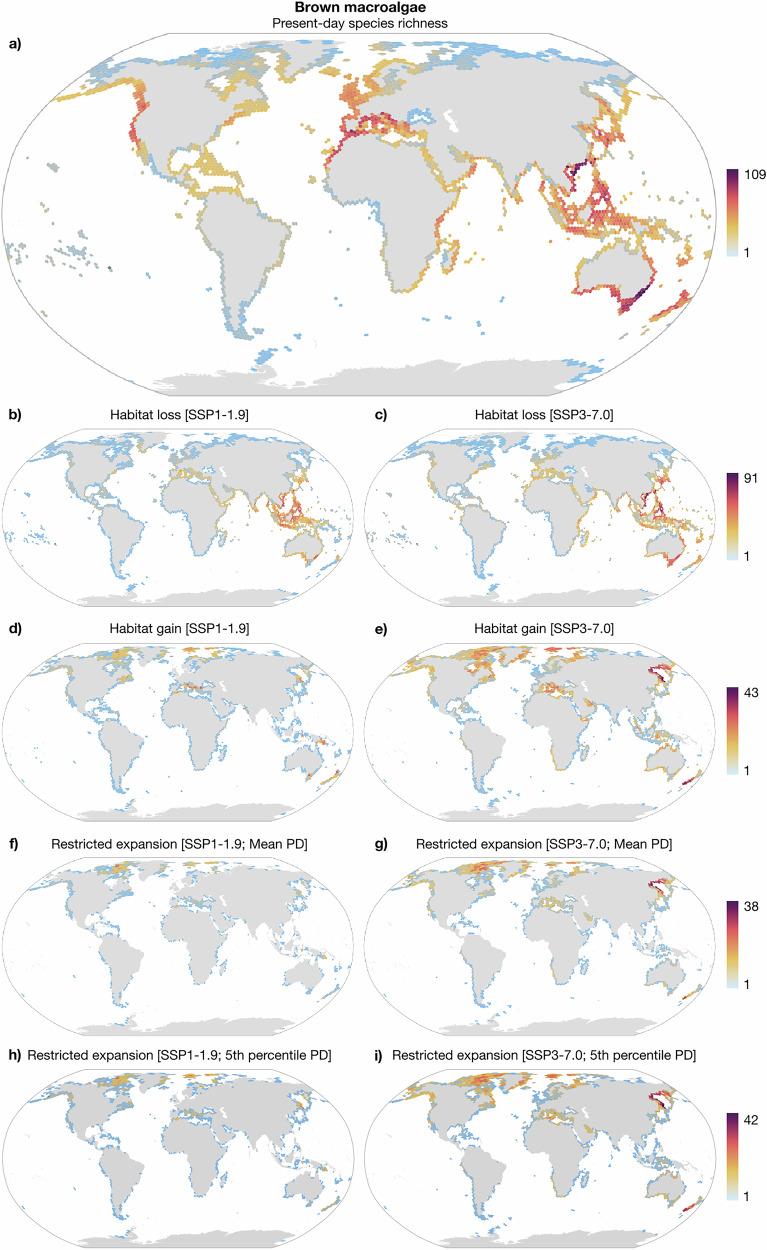
Projected range shifts of brown macroalgae under contrasting Shared Socioeconomic Pathway (SSP) scenarios of climate change. Present-day patterns of **a** species richness and projected habitat **b**, **c** losses and **d**, **e** gains under climate change. Regions of restricted range expansion to newly suitable habitats owing to the effect of oceanographic connectivity under the **f**, **g** mean and the **h**, **i** 5th percentile of propagule duration. Maps aggregated to a coarser resolution of 150 km for better visualization (refer to data availability statement for high-resolution maps).

Present-day biodiversity hotspots for seagrasses (>15 species) were concentrated in the Indo-Pacific, particularly within the Coral Triangle and the South China Sea, as well as along the West African, Northwest and Southeast Australian coastlines (Fig. 2). Brown macroalgae hotspots (>50 species) also included the Indo-Pacific and Australia, as well as the West Mediterranean/adjacent Atlantic, British Isles, and the Northwest Pacific Ocean (Fig. 3). Under projected climate change, the Indo-Pacific is projected to face the most pronounced habitat losses, with the China Sea, Philippine Sea, Java Sea, and along the Eastern Australian coastline potentially losing more than 10 seagrass species (Fig. 2) and more than 40 brown macroalgae species (Fig. 3). Additional moderate losses extend across the tropical Western Pacific Ocean. Without dispersal limitations, the Okhotsk Sea, southern Australia, New Zealand, and southern Angola coastlines showed the greatest potential for seagrass range expansions (Fig. 2). For brown macroalgae, unrestricted range expansions are higher in the Okhotsk Sea and New Zealand. Additional areas of moderate expansions include the temperate Northeast Pacific, the Arctic, the central Mediterranean Sea, the Persian and Aden Gulfs (Fig. 3). Oceanographic connectivity, both under the mean and the 5th percentile of propagule duration, is expected to limit future range expansions in the Okhotsk Sea and southern New Zealand. Specifically for seagrass, additional limitations to range expansions were identified in southern Australia and southern Angola, while for brown macroalgae in the Arctic, and to a lesser extent in the central Mediterranean, and the temperate Northeast Pacific (Figs. 2 and 3).

## Discussion

Our study demonstrates that oceanographic connectivity is a major limiting factor in the climate-induced range shifts of marine forests. By integrating species distribution and biophysical models, we show that while there is potential for marine forests to shift their distributions poleward and partially offset range losses, well-defined dispersal barriers created by ocean currents emerge as a critical bottleneck, significantly constraining these movements. Under the most conservative scenarios, these barriers can reduce expansions into future suitable habitats by 51.93% for seagrasses and 71.75% for brown macroalgae, leaving regions with broad suitable habitats in the future potentially uncolonized. This restriction on dispersal has profound implications for the overall net change in suitable habitats, increasing the proportion of species facing negative impacts. These findings underscore the critical need to consider projected habitat shifts alongside oceanographic connectivity when assessing the vulnerability and resilience of marine biodiversity under climate change.

SDM reveals present-day biodiversity hotspots for seagrasses in the Indo-Pacific, West Africa, and Australia, while for brown macroalgae in the Indo-Pacific, Australia, Northeast Pacific, west Mediterranean/adjacent Atlantic, and along the British Isles. These estimates at the regional scale align with previous studies and reflect established evolutionary hypotheses of species radiation and diversification^10,23,24^. Future projections indicate substantial range contractions for both groups, particularly under the high emissions scenario (SSP3-7.0), a pattern consistent with projections performed for additional taxa^10,25,26^. The Indo-Pacific region is a particularly vulnerable area, with severe declines in the East China Sea, Philippine Sea, Java Sea, and along the Eastern Australian coastline. Additional moderate contractions are projected across tropical and temperate regions globally. These range losses, adding up to ongoing declines reported for marine forests^27–29^, threaten to disrupt essential ecosystem services, such as carbon sequestration, coastline protection, and the provision of nursery grounds and habitat for diverse marine life^13,14,30,31^. Even if functionally similar species replace the lost ones, maintaining regional levels of biodiversity, cascading effects on community composition and trophic interactions can still occur, compromising overall ecosystem health and resilience^32^.

Without dispersal limitations, SDMs suggest wide poleward range expansions into newly suitable habitats. Hotspots of range expansions include the Okhotsk Sea, southern Australia, and New Zealand, as well as additional areas like southern Angola coastlines, the temperate Northeast Pacific, the Arctic, the central Mediterranean Sea, and the Persian and Aden Gulfs. Despite potential ecological shifts in these regions, with new species established impacting community composition and trophic interactions^33^, range expansions can partially offset the negative impacts of range losses at the species level. However, oceanographic connectivity is shown here as a crucial factor restricting expansions and, therefore, negatively impacting distribution net changes under climate change. Previous studies hypothesized that oceanographic connectivity may modify the coupling between climate change and biogeographical shifts^4^. Our study is the first to quantify the extent to which this connectivity may restrict species range displacements in the ocean. This limitation is particularly pronounced under higher emissions, and its magnitude is strongly dependent on the dispersal scenario considered. Crucially, moving from the mean to the conservative 5th percentile of propagule duration increases the restriction on range expansions by 31.40% for seagrasses and by 49.82% for brown macroalgae. Under such restricted dispersal conditions, populations may become strongly isolated, unable to track shifting climates, ultimately increasing their risk of extinction. Vast regions of future suitable habitats, like the Okhotsk Sea, southern New Zealand, southern Angola, the Arctic, the Mediterranean, and the temperate Northeast Pacific, may remain uncolonized due to dispersal barriers created by oceanographic connectivity.

Oceanographic connectivity may have strong implications for future range shifts; however, restrictions on range expansions were on the same order of magnitude for both seagrasses and brown macroalgae, despite variations in propagule duration periods (both between groups and between the mean, 5th, and 95th percentile estimates). This suggests that ocean currents create strong dispersal barriers that even longer-lived propagules cannot reliably cross^34^. These barriers may thus apply to additional passively dispersed marine taxa. The impact of these barriers is evident in the observed multigenerational stepping-stone connectivity, which averaged up to 15.23 events for seagrasses and 12.60 events for brown macroalgae, far below the simulated potential of 80 events, which aimed at ensuring that every candidate colonization sequence could unfold within the 2020–2100 period. This aligns with previous studies demonstrating the skewed, non-linear relationship between ocean currents and population connectivity^6^, which often results in significant genetic differentiation levels, even for species with high dispersal potential^35–37^. Correspondingly, oceanographic connectivity can impact future diversification and speciation processes^34,38,39^. Where dispersal barriers consistently restrict population connectivity, vicariant processes can drive the divergence of once-homogeneous genetic lineages^34,38^. Conversely, highly connected populations can interbreed through time, increasing their genetic diversity levels and, therefore, their resilience and adaptability potential under climate change^40^ (but see outbreeding depression^41^). Where future range expansions are supported, founder effects at the leading edge of colonization can significantly reshape genetic structures^42^, particularly well reported for marine forest species^43,44^. Recognizing the complex interaction between oceanographic connectivity and evolutionary processes is key to anticipating the resilience and adaptive capacity of marine biodiversity in the face of climate change.

Integrating BM and SDM estimates has shown to provide valuable insights into the role of oceanographic connectivity in shaping potential range dynamics under climate change. However, it is crucial to acknowledge a set of inherent uncertainties. First, the BM integrates ocean current data at a resolution of 1/16° ^45^, meaning that finer-scale nearshore processes might be unresolved^46^. These can be important for short-distance dispersal events (e.g., seeds and spores), reshaping local connectivity patterns^47,48^. Second, the current velocity fields utilized in BM are restricted to the surface of the ocean, potentially underestimating the connectivity of deep cryptic populations. While most marine forests are found within the mixed layer depth (down to ~45 meters), some species (e.g., *Laminaria ochroleuca* or *Macrocystis pyrifera*) can thrive in offshore seamount environments, where clear, nutrient-rich water extends their distributions to greater depths^34,49^. There, deeper currents may shape different dispersal patterns than those predicted by the surface-based model. Third, the BM is based on present-day ocean currents. While future climate change scenarios project shifts in ocean circulation that may alter dispersal pathways and barriers^50^, analyses are constrained by data resolution. Earth System Models used to project changes in ocean currents have a coarse resolution that cannot yet resolve the critical nearshore oceanographic processes necessary for accurate coastal dispersal modeling^51,52^. Fourth, the highly variable propagule duration data available in the literature, along with gaps for many species in our dataset (31 species with data for a total of 467), further introduces uncertainty. The use of mean and 95^th^ percentile durations aimed at providing a general perspective of dispersal kernels^53,54^. However, propagule durations are species-specific (Supplementary Information), potentially leading to an underestimation of dispersal limitation. The inclusion of the most restrictive 5th percentile PD scenario provides a conservative estimate of range shift limitations, directly addressing the potential for high early-stage mortality^55^, as well as low propagule durations linked to high settling velocity or reduced rafting potential^35,56,57^. The variability and uncertainty related to species dispersal potential highlight the need for further research to refine modeling frameworks^58^. Finally, the SDM were built strictly with climatic variables, neglecting the availability of suitable substrate (e.g., sandy bottoms for seagrass and rocky reefs for macroalgae), and the potential for important biotic interactions (e.g., competition for available space^59^), which together can further influence the ability of species to colonize newly suitable habitats^3^. However, detailed, high-resolution global datasets on substrate type or biotic interactions are not currently available on a global scale. Integrating such data remains a major challenge for global-scale projections of marine biodiversity. It is important to consider these uncertainties when interpreting the model outputs, particularly if these are considered in conservation strategies under a changing climate.

Overall, our findings highlight the need for a paradigm shift in marine biodiversity assessments and conservation strategies to effectively address the challenges of climate-driven species redistribution. Planning the future based solely on projected habitat suitability (i.e., climate-smart conservation^60^) might be insufficient, as oceanographic connectivity may significantly restrict range expansions for marine species. These results have strong implications for climate-adaptation and mitigation of activities that depend on marine resource utilization, as human populations along vast coastlines across the globe base their subsistence highly on marine biodiversity associated with marine forests. Furthermore, the important role played by oceanographic connectivity demonstrates that scales of biodiversity management go beyond political borders, as even the best local management practices can be ineffective if along oceanographic dispersal pathways external threats block connectivity. For conservation, we emphasize the importance of incorporating connectivity estimates into SDM-based marine conservation planning; a feature abundantly recognized in terrestrial conservation^61^. For instance, marine protected areas (MPAs) strategically placed within, or along future dispersal pathways, can safeguard species movements under climate change^62^. On the other hand, prioritizing areas where ocean currents create major dispersal barriers can help mitigate the risk of isolation and potential extinction for vulnerable populations^63^. By integrating knowledge of oceanographic connectivity alongside habitat suitability^64^ conservation strategies can be tailored to promote assisted colonization efforts, restoration initiatives^65^, and the design of well-connected networks of MPAs^66^, in line with the 2020 Global Biodiversity Framework aimed at protecting 30% of the oceans by 2030^67,68^. Overall, our findings underscore the urgent need for a paradigm shift in marine biodiversity assessments and conservation strategies to effectively address climate-driven species redistribution.

## Methods

### Theoretical framework

To assess how oceanographic connectivity might limit the range shifts of seagrasses and brown macroalgae under future climate change, we developed a framework that combines species distribution models (SDMs) with biophysical connectivity models (BMs). We used the SDMs to project shifts in suitable habitats from present-day conditions to the end of the century under various climate scenarios. Concurrently, we used BMs to simulate species’ dispersal potential, using long-term ocean current patterns and propagule durations (PDs). By comparing scenarios of restricted and unrestricted dispersal, our framework quantifies the extent to which marine forests may be unable to colonize newly suitable habitats.

### Species distribution models

Species distribution maps of 58 seagrass species (order Alismatales; Supplementary Data 1) and 409 brown macroalgae species (orders Chordales, Desmarestiales, Fucales, Laminariales, and Tilopteridales; Supplementary Data 1) under present-day conditions (decade 2010-2020) and end-of-century (decade 2090–2100) Shared Socioeconomic Pathway (SSP) scenarios of future climate change were acquired from a published dataset^69^. This dataset provides distribution maps at 0.05 degrees resolution (~5 km) based on high-performance SDM generated with an ensemble of advanced machine learning techniques, which fitted expert-curated species’ occurrence records^70^ against biologically relevant, high-resolution environmental variables^51^, such as ocean temperature, nutrient conditions (e.g., nitrate), sea ice cover, wave energy, and salinity. To consider the potential range of future changes, we considered the lower emission scenario of sustainable development, aligned with the Paris Agreement (SSP 1-1.9), and the high emission scenario of inequality development (SSP3-7.0). Comparing the habitat suitability maps for the different time periods and climate scenarios allowed identifying regions of range loss and gain per species. Specifically for range gains, we further determined the distance between present-day suitable habitats and the nearest future suitable habitats. Additionally, the number of species that might gain or lose suitable habitats was determined per ocean cell for the two SSP scenarios^10^.

### Biophysical connectivity models

Oceanographic connectivity estimates were acquired from coastalNet, a dataset^45^ providing BM predictions of global dispersal pathways. The dataset was built by simulating population connectivity of passively dispersed species using 21 years of daily data on the direction and intensity of ocean currents derived from the Global Ocean Physics Reanalysis (EU Copernicus Marine Service)^35^. The main output of the BM is probabilities of connectivity between coastal sites globally, which have been widely benchmarked against population genetic and demographic differentiation patterns for numerous marine forest species^6,12,34,35^.

Seagrass and brown macroalgae dispersal stages comprise propagule durations that can vary considerably^34,53,71^. To incorporate this variability into the BM, a database of empirical propagule duration records was acquired from literature^53,54^. This database comprises 31 species (13 seagrasses and 18 brown macroalgae; Supplementary Information)^55,71^. Due to the scarcity of species-specific data, we summarized the database into the 5th percentile, the mean, and the 95th percentile of propagule duration for each group (e.g. ^53,54^), thereby providing a comprehensive gradient of dispersal potential. The 5th percentile PD serves as a conservative scenario; the mean PD represents the most frequent, typical cross-species dispersal potential; and the 95th percentile PD captures the less common events associated with episodic long-distance dispersal^71^. Seagrasses exhibited a mean propagule duration of 12.53 ± 8.12 days (5th percentile of 2.60 days and 95th percentile of 24.80 days), while brown macroalgae exhibited a longer mean propagule duration of 19.94 ± 35.74 days (5th percentile of 0.85 days and 95th percentile of 59.05 days; Supplementary Information).

The connectivity estimates were assembled into a global, coast-wide directed graph (i.e., network analysis/graph theory). Each node corresponds to a coastal site, and its edges to the probability of a marine forest propagule dispersing from a source site to a destination site within the group-specific mean and 95th percentile propagule-duration windows. The graph was filtered per species to determine the connectivity potential from sites with present-day suitable habitats to sites with newly end-of-century habitats. These pathways were found using Dijkstra’s algorithm, and the number of intermediate sites within each path was counted; this count represents the number of “stepping-stones” required for whole colonization pathways across generations. Kelp and seagrass species typically reach reproductive maturity within a single growing season - usually in less than one year after establishment^72–75^. We therefore treat one stepping-stone as 1 year, and retain only colonization pathways comprising ≤ 80 steps, ensuring that every candidate colonization sequence could unfold within the 2020–2100 simulation window, matching the SDM projections.

### Impact of oceanographic connectivity on species range shifts

**Fig. 4:**
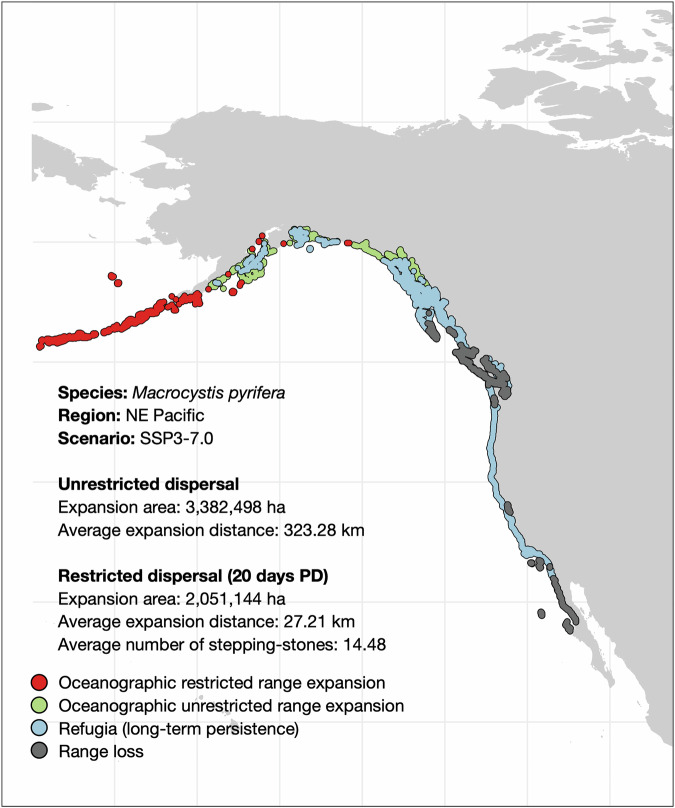
Example of the influence of oceanographic connectivity in projected range shifts of the brown macroalgae Macrocystis pyrifera under the Shared Socioeconomic Pathway (SSP) scenario 3-7.0 of higher emissions. Comparison of unrestricted versus restricted dispersal by oceanographic connectivity using a propagule duration period of 20 days, within the reported range of the species’ dispersal potential^56^^,^^71^.

To quantify the relative impact of oceanographic connectivity on species range expansions, we compared unrestricted versus restricted by oceanographic connectivity (refer to example on Fig. 4). Specifically, we fitted a linear mixed-effects models (LMM) with (1) range expansion in area, (2) range expansion in distance, (3) net change in range expansion in area, and (4) number of stepping-stones used across putative colonization pathways as model responses, taxonomic group (seagrass vs. brown macroalgae) and scenario (SSP1-1.9 vs. SSP3-7.0) as fixed effects, and a random intercept to account for repeated measures across species. Tukey tests (*p*-values adjusted) were used to determine the significance of multiple pairwise comparisons.

## Supplementary information

Supplementary information

Supplementary data 1

Supplementary data 2

Supplementary data 3

Supplementary data 4

## Data Availability

The range maps used to produce the results are permanently available at:10.6084/m9.figshare.27075493.

## Code availability

The code used to produce the results is permanently available at: 10.6084/m9.figshare.27075493.
