# Supplementary material for: Oceanographic connectivity strongly restricts future range expansions of critical marine forest species
Source: NPJ Biodivers. 2026 Mar 27;5:10. doi: 10.1038/s44185-026-00123-y (PMC13031816; doi:10.1038/s44185-026-00123-y)
Supplement: Supplementary file 1 — Supplementary information [file 44185_2026_123_MOESM1_ESM.pdf]

Supplementary Information

Oceanographic connectivity strongly restricts future range expansions of critical marine forest species

Jorge Assis, Eliza Fragkopoulou, Ester A. Serrão, Miguel B. Araújo

Supplementary information. Distribution of propagule duration periods of marine forest species and average propagule duration per species.

Supplementary Data 1. Empirical information from published literature on propagule duration periods of seagrass and brown macroalgae species.

Supplementary Data 2. Influence of oceanographic connectivity in projected range shifts of seagrasses and brown macroalgae species under contrasting Shared Socioeconomic Pathway (SSP) scenarios of climate change.

Supplementary Data 3. Linear mixed-effects models and Tukey tests to determine the significance of range shift differences between taxonomic groups (seagrass and brown macroalgae), climate change scenarios and dispersal restrictions.

Supplementary Data 4. Summary of the influence of oceanographic connectivity in projected range shifts of seagrasses and brown macroalgae under contrasting Shared Socioeconomic Pathway (SSP) scenarios of climate change.

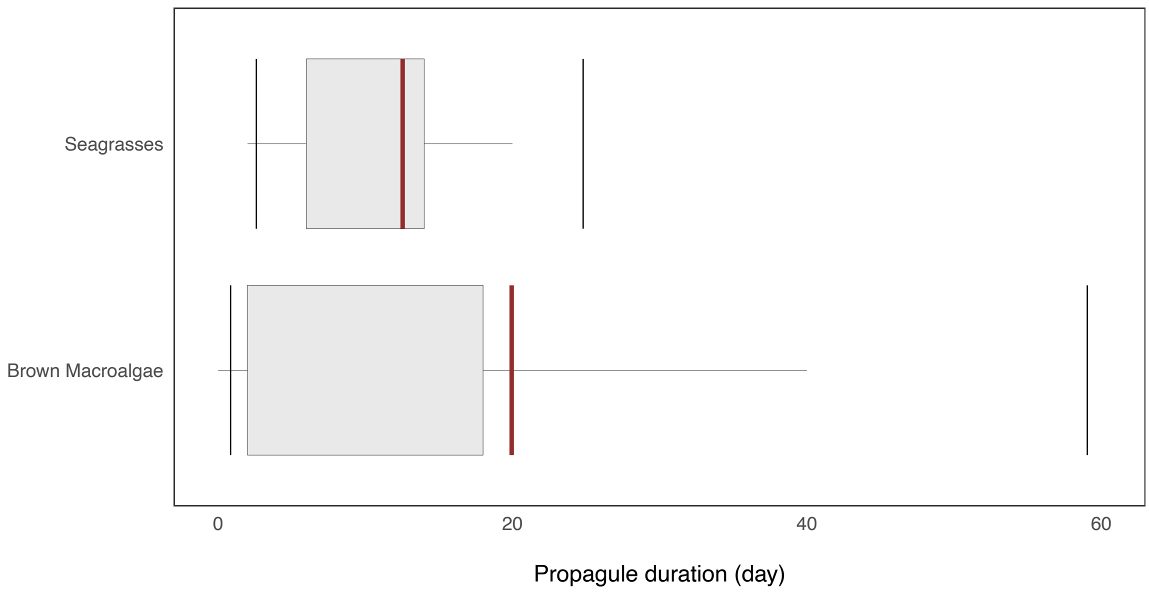

Fig. S1. Distribution of propagule duration periods of marine forest species. Database acquired from literature [1,2]. Vertical lines depict the mean (red) and the extreme 5th and 95th percentiles (black) of PD observations across observations. Seagrasses exhibited a mean propagule duration of 12.53± 8.12 days (5th percentile of 2.60 days and 95th percentile of 24.80 days), while brown macroalgae exhibited a longer mean propagule duration of 19.94± 35.74 days (5th percentile of 0.85 days and 95th percentile of 59.05 days).

Table S1. Average propagule duration per species and corresponding references (DOI: Digital Object Identifier). Database acquired from literature [1,2].

scientificname	PD	DOI
Alaria marginata	2	http://dx.doi.org/10.3354/meps260083
Ascophyllum nodosum	43	https://doi.org/10.1016/S0022-0981(98)00089-6
Cymodocea rotundata	20	https://doi.org/10.3389/fpls.2018.00160
Durvillaea antarctica	40	10.1098/rspb.2010.1117
Ecklonia maxima	150	https://doi.org/10.1098/rspb.2014.0878
Enhalus acoroides	6	http://dx.doi.org/10.3354/meps235075
Fucus distichus	1	10.3354/meps300063
Fucus vesiculosus	0	http://dx.doi.org/10.3354/meps09238
Halodule uninervis	18	https://doi.org/10.3389/fpls.2018.00160;
Halodule wrightii	14	http://dx.doi.org/10.3354/meps310109
Halophila johnsonii	4	http://dx.doi.org/10.3354/meps310109
Halophila ovalis	14	https://doi.org/10.3389/fpls.2018.00160;
Hormosira banksii	37	10.1111/j.1529-8817.2008.00563.x
Laminaria digitata	3	http://dx.doi.org/10.3354/meps09238
Laminaria hyperborea	1	10.1017/S0025315400024929
Laminaria ochroleuca	5	https://doi.org/10.1111/jbi.13425
Laminaria pallida	7	https://doi.org/10.1111/jbi.14338
Lessonia nigrescens	2	10.1016/0022-0981(89)90193-14
Macrocystis pyrifera	9	10.1016/S0022-0981(00)00255-0; 10.1890/0012-
Posidonia australis	32	https://doi.org/10.1098/rspb.2014.0878;
Posidonia oceanica	12	https://doi.org/10.1098/rspb.2014.0878
Posidonia sinuosa	2	https://doi.org/10.1098/rspb.2014.0878
Pterygophora californica	1	10.2307/1940011
Saccorhiza polyschides	5	https://doi.org/10.1111/jbi.13425
Sargassum muticum	18	10.1016/0022-0981(81)90188-X
Sargassum ringgoldianum	32	10.2331/SUISAN.33.923
Thalassia hemprichii	14	https://doi.org/10.1371/journal.pone.0156585;
Thalassia testudinum	3	https://doi.org/10.1098/rspb.2014.0878; https://doi.org/10.1525/bio.2012.62.1.10;
Undaria pinnatifida	3	10.2216/i0031-8884-39-6-547.1
Zostera marina	11	https://doi.org/10.1098/rspb.2014.0878; https://doi.org/10.2307/1941597;
Zostera noltii	13	https://doi.org/10.1098/rspb.2014.0878

References

1. Assis, J. *et al.* Weak biodiversity connectivity in the European network of no-take marine protected areas. *Science of the Total Environment* **773**, 1–24 (2021).

2. Assis, J. *et al.* Potential Biodiversity Connectivity in the Network of Marine Protected Areas in Western Africa. *Front Mar Sci* **8**, 1749 (2021).
